# P06 Drip, drip, hooray! A deep dive into outpatient parenteral antimicrobial therapy awareness at an Irish university hospital

**DOI:** 10.1093/jacamr/dlaf239.010

**Published:** 2026-01-05

**Authors:** Mubashir Habib, James Lalor O Neill, Kate Moran, Ashwin Delmonte Sen, Sinead Fenlon, Aoife Pearson, Sarmad Waqas

**Affiliations:** Infectious Diseases Department, Tallaght University Hospital, Dublin, Ireland; Infectious Diseases Department, Tallaght University Hospital, Dublin, Ireland; Infectious Diseases Department, Tallaght University Hospital, Dublin, Ireland; Infectious Diseases Department, Tallaght University Hospital, Dublin, Ireland; Infectious Diseases Department, Tallaght University Hospital, Dublin, Ireland; Infectious Diseases Department, Tallaght University Hospital, Dublin, Ireland; Infectious Diseases Department, Tallaght University Hospital, Dublin, Ireland; Clinical Medicine TUH, School of Medicine, Trinity College Dublin, Dublin, Ireland

## Abstract

**Background:**

Outpatient parenteral antimicrobial therapy (OPAT) enables safe administration of IV antimicrobials in the community, reducing hospital stay and improving antimicrobial stewardship. However, awareness and implementation can be variable across different institutions.

**Objectives:**

This study aimed to assess awareness, familiarity, and confidence regarding OPAT protocols amongst healthcare professionals in a university hospital, and to identify perceived barriers to OPAT utilization.

**Methods:**

A cross-sectional, anonymized study was conducted in September 2025 among staff at an Irish university teaching hospital following approval from the Quality Improvement (QI) department (ID: 5479). Data was collected using a structured questionnaire administered online. Participants included consultants, registrars, senior house officers , interns, and various grades of nurses. The questionnaire assessed OPAT awareness, perceived benefits and contraindications, knowledge of governance, confidence in OPAT assessment, and participant’s perceived need for further training.

**Results:**

A questionnaire was sent to 800 healthcare professionals, of whom 136 opened the link and 74 responded. The composition of respondents was: consultants 16% (*n*=12), registrars 22% (*n*=16), clinical nurse managers 22% (*n*=16), senior house officers 15% (*n*=11), interns 16% (*n*=12), and others (including nurse practitioners) 9% (*n*=7). Most respondents (46%, *n*=34) had more than 10 years of healthcare experience, with 18% (*n*=13) reporting 6–10 years, 20% (*n*=15) 1–5 years, and 16% (*n*=12) less than 1 year. Overall, 62% (*n*=45) had previously used OPAT, while 36% (*n*=26) were aware of OPAT but had never used it, and 3% (*n*=2) were not aware of OPAT. The infectious diseases team was correctly identified as the OPAT service’s clinical governance speciality by 82% (*n*=58) of respondents. The most frequently perceived benefits of OPAT were reduced hospital stay (99%, *n*=72), improved patient comfort (97%, *n*=71), reduced healthcare-associated infection risk (92%, *n*=67), cost savings (89%, *n*=65), and improved treatment compliance (79%, *n*=58). Confidence in identifying OPAT-appropriate patients was variable: 19% (*n*=14) reported being very confident, 61% (*n*=44) somewhat confident, and 19% (*n*=14) not confident. Among non-users or those only aware of OPAT (*n*=37), barriers included unclear referral pathways (30%, *n*=11), previous bad experiences (8%, *n*=3), and availability of alternative outpatient antibiotic pathways (11%, *n*=4), while 51% (*n*=19) reported other or no barriers. A large majority (85%, *n*=63) felt that additional formal OPAT training would be beneficial. Participants were provided with correct answers at the end of the questionnaire, so the survey also functioned as both an assessment of awareness as well as an educational tool.

**Conclusions:**

Awareness of OPAT was high among healthcare professionals in our institution, with strong recognition of its clinical and economic benefits. However, confidence in patient selection and uncertainty regarding referral pathways were identified as barriers to wider adoption. Many respondents felt that more formal OPAT training is needed. These findings will inform future OPAT programme expansion and education strategies.

